# Longitudinal Alterations of Retinal and Choroidal Structure in Patients Recovered from COVID-19

**DOI:** 10.1155/2022/4123328

**Published:** 2022-03-25

**Authors:** Mojtaba Abrishami, Kiana Hassanpour, Ghodsieh Zamani, Seyedeh Maryam Hosseini, Nasser Shoeibi, Zahra Emamverdian, Amir Zamani, Nasibeh Amini, Majid Abrishami

**Affiliations:** ^1^Eye Research Center, Mashhad University of Medical Sciences, Mashhad, Iran; ^2^Ophthalmic Research Center, Research Institute for Ophthalmology and Vision Science, Shahid Beheshti University of Medical Sciences, Tehran, Iran; ^3^Department of General Surgery, Loghman Medical Center, Shahid Beheshti University of Medical Sciences, Tehran, Iran

## Abstract

**Objective:**

To evaluate the midterm longitudinal changes in chorioretinal structures in patients with coronavirus disease 2019 (COVID-19).

**Methods:**

Thirty-four eyes of 17 COVID-19 patients were enrolled. The patients underwent retinal and choroidal imaging upon the recovery (baseline) after 1 and 3 months. Retinal measurements in fovea, parafovea, and perifovea were recorded. To calculate choroidal vascularity index (CVI), luminal and total choroidal areas were measured using Sonada's method. Choroidal thickness was measured at the subfovea 500 microns temporal and nasal to the fovea.

**Results:**

Mean CVI was 0.64 ± 0.04 at baseline that significantly increased to 0.67 ± 0.05 (*P* = 0.012) after 1 month and again significantly decreased to 0.63 ± 0.05 after 3 months (*P* < 0.001). While the stromal component showed a significant decrease between the baseline and first-month values (1.16 ± 0.29 to 1.01 ± 0.27, *P* = 0.03), the luminal area mostly changed between months 1 and 3 (2.03 ± 0.28 to 1.91 ± 0.23, *P* = 0.045). The average of subfoveal choroidal thickness and retinal thickness remained unchanged.

**Conclusion:**

CVI is increased in patients with COVID-19 1 month after recovery from COVID-19 and returns to baseline values after 3 months. Regarding the reversible nature of changes, there might be a prominent role in inflammation.

## 1. Introduction

Since the first emergence of the severe acute respiratory syndrome coronavirus 2 (SARS-CoV-2), various extrapulmonary manifestations of the disease have been reported [1]. Although the initial reports mainly focused on the pulmonary and systemic involvement of the virus, later studies investigated less common signs, including ocular manifestations [2].

In patients with coronavirus disease 2019 (COVID-19), various retinal manifestations have been reported to date [3–6]. Marinho et al. [7] first described the involvement of retinal microcirculation that leads to microhemorrhages and cotton wool spots. While the validity of these findings has been questioned by attributing the lesions to the normal retinal vessels [8], later studies also confirmed the retinal microvascular changes [4, 9]. In addition to retinal manifestations, choroid can also be involved during the disease course [10, 11].

Multiple mechanisms have been proposed to explain the retinal involvement during COVID-19, including the treatment or drug effects, the concomitant risk factors, inflammatory markers, and the direct invasion of the virus [12]. Presence of angiotensin-converting enzyme 2 (ACE2) receptors, the primary receptor in the pathogenesis of COVID-19, in retina and choroid has been confirmed by both cadaver and experimental studies [13–15]. However, the exact mechanisms that lead to retinal changes are still unclear.

Choroidal vascularity index (CVI) is a newly diagnosed marker to study the vascularity of choroid in different retinal pathologies [16–19]. Recent study by Agarwal and associates proposed that CVI can serve as a more stable index to show the changes of the choroid in comparison to subfoveal choroidal thickness (SFCT) since CVI is less affected by physiologic variables like axial length, age, intraocular pressure, and systolic blood pressure [16]. Also, CVI has less variability than SFCT. On the other hand, the choroidal structure is mainly vascular and ocular pathologies that affect the choroid work through vascular changes [20]. To better elucidate the vascular changes of choroidal tissue, CVI can be a more direct surrogate marker than measuring SFCT.

To better understand the pathophysiological aspects of the chorioretinal changes in COVID-19, longitudinal studies are needed to follow the changes. To the best of our knowledge, no longitudinal study reports the serial changes in the retina and choroid. The present study aimed to investigate the retinal and choroidal structural changes in patients with COVID-19.

## 2. Methods

### 2.1. Study Population

In a prospective observational study, patients with a definite history of COVID-19 confirmed by a positive nasopharyngeal swab sample real-time, reverse transcription-polymerase chain reaction (RT-PCR) result, and a recovery history from the systemic symptoms for at least one week were included. Detailed ocular and systemic records were obtained from each subject. Patients recruited for this study were all personnel of Khatam Eye Hospital, who had recovered from COVID-19, and all volunteered to undergo ophthalmological and ocular imaging examination for research investigation. All the patients were outpatient, and none of them received systemic corticosteroids.

Exclusion criteria included any history of refractive or intraocular surgery, any history of clinically apparent retinal or choroidal diseases, or glaucoma. Any patients who admitted a history of diabetes mellitus, autoimmune disease, current pregnancy, breastfeeding, or migraine were also not enrolled. Additional exclusion criteria included absolute spherical refractive error greater than 5 diopters and cylindrical refractive error of more than 2 diopters. Any evidence of ocular media opacity preventing high-quality imaging or reduced OCT scan quality was also excluded from the analysis. Any subjects with the best-corrected visual acuity less than 20/20 were also not included in the protocol. Patients with a history of systemic corticosteroid therapy were also excluded.

Complete history regarding the patients' symptoms, disease course, and hospitalization was recorded. Refraction was evaluated using a KR-1 Auto Kerato-Refractometer (Topcon Medical Systems, Inc., Tokyo, Japan)

### 2.2. Retinal and Choroidal Imaging

Using complete protection by personal protective equipment (PPE), an experienced operator took images of the patients. To evaluate retinal total, inner, and outer thickness, the AngioVue (RTVue XR Avanti, Optovue, Fremont, CA, USA; software version 2018.0.0.14) system Raster and Retina Map protocols were used. Images with a signal strength of less than 45 were retaken. The Heidelberg Spectralis device (Heidelberg Engineering, Heidelberg, Germany) with enhanced depth imaging optical coherence tomography (EDI-OCT) was used to evaluate the choroid. Automatic Real-Time function (ART) was set 9–15, and the images with quality of less than 20 were retaken. Method of image acquisition has been previously reported [21]. To minimize the variation of CVI and choroidal thickness, all images were taken between 9 and 11 a.m. Patients were visited 1 month and 3 months after the first visit to repeat imaging.

Retinal parameters included retinal thickness in the whole image, fovea, parafovea, inner retina, and outer retina.

### 2.3. Image Processing

Choroidal thickness (CT) was measured using ImageJ software (National Institute of Health, Bethesda, MD, USA). CT was manually measured at the subfoveal, 500 µm nasal, and 500 µm temporal to the fovea. Subfoveal choroidal thickness was measured between the outer portion of the hyperreflective line of retinal pigment epithelium (RPE) and the inner portion of the choroidoscleral junction at the fovea.

**Figure 1:**
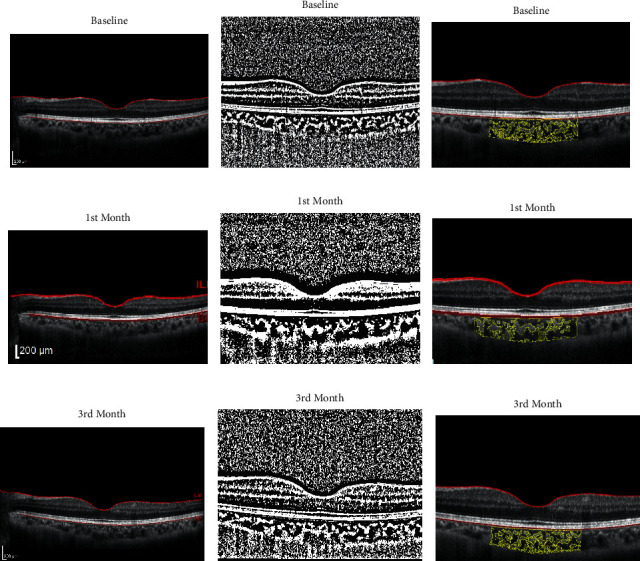
Choroidal vascular index (CVI). The first column (a–c) shows the baseline visit of a patient. The second (d–f) and third (g–i) columns relate to the same person in the first and third visits. To calculate CVI, the image was converted to 8 bits and the brightness was set at the measure of the average of three 9-micron lumens of blood vessels. Corneoscleral junction was determined. The image was binarized using Niblack auto local threshold (the middle rows). The area was added to the ROI tool. Then, the image was converted to the RGB, and the color threshold tool was used for thresholding the image. The area was added to ROI; another time was with the AND tool. The selected area was measured as the luminal area (LA). In the last rows between the yellow areas, the grey zones represent the LA and the bright zones demonstrate the stromal area. The CVI was calculated by dividing LA by the total choroidal area.

CVI was calculated using Sonada's method [22] with ImageJ software. Briefly, the image was opened in ImageJ. The image was converted to 8 bits, and the contrast was set at the measure of the average of three 9-micron darkest lumens of the blood vessel using the oval tool. Corneoscleral junction was determined. The scale of the image was defined using a set scale. The area of 1500 µm nasal and temporal to the fovea was selected using the polygonal tool, and the total choroidal (TCA) area was measured. TCA was the area between the outer edge of hyperreflective area in superior, sclerocorneal junction in inferior, 1500 µm nasal and temporal to the fovea. The area was added to the ROI tool. The image was binarized using Niblack autolocal threshold. Then, the image was converted to the RGB, and the color threshold tool was used for thresholding the image. The area was added to ROI; another time the AND tool was applied. The selected area was measured as the LA. The CVI was calculated by dividing LA to the TCA (Figure 1).

To assess interrater reliability, 2 graders (KH, AZ) measured the first visits of all patients. In the same way, to evaluate intraobserver reliability, measurements of patients' first visit were repeated 1 week after the first calculation. Intraclass correlation coefficient (ICC) and Bland–Altman plots were used to evaluate the agreements. After achieving good agreement, all measurements were performed by one grader (KH), who was masked from the timing of the patients' examinations.

### 2.4. Statistical Analysis

The mean, median, standard deviation, and interquartile range were used to describe the data. The normality of the data was tested using Kolmogorov–Smirnov test. The generalized estimating equation was used for repeated measure analysis (within-subject changes). Cronbach's alpha was used to report the interrater and intrarater agreements. All analyses were performed using SPSS version 25 (IBM Corp., Armonk, New York, USA). A *P* value less than 0.05 was considered as the significance level.

## 3. Results

A total of 34 eyes of 17 COVID-19 patients (10 females, 58.8%) with a mean age of 34.6 ± 6.7 years were enrolled in the study after applying exclusion criteria.

### 3.1. Quantitative Retinal Changes

Retinal parameters, including thickness and volume at fovea, parafovea, and perifovea, remained comparable to baseline values after 1 and 3 months (Table 1). There was an increase in outer retina thickness in both parafovea and perifovea. Thickness in the whole image of the outer retina increased from 179.5 ± 6.7 at baseline to 181.3 ± 7.2 after 1 month and 184.3 ± 8.1 after 3 months. The corresponding values in perifovea were 170.1 ± 6.9, 171.3 ± 6.6, and 174.4 ± 8.4, respectively. However, this increasing pattern did not reach a significant level. Of note, the values in the inner retinal did not show any increasing pattern.

### 3.2. CVI and Choroidal Thickness

**Figure 2:**
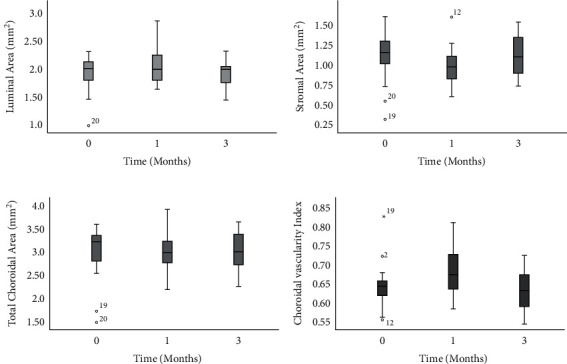
Boxplots demonstrating luminal area (a), stromal area (b), total choroidal area (c), and choroidal vascularity index (d) over the study period. Zero-point indicates recovery from the disease, and 1 and 3 indicate 1 and 3 months after the first visit. The luminal area significantly changed between 1 and 3 months, while the stromal area demonstrated significant changes between baseline and first month. The total choroidal area remained comparable between the study periods while CVI increased significantly between the baseline and first month and then returned to baseline values after 3 months.

Mean of CVI was 0.64 ± 0.04, at baseline (median = 0.64, range = 0.27), which significantly increased to 0.67 ± 0.05 (median 0.66, range = 0.17, *P*=0.012) after 1 month and again significantly decreased to 0.63 ± 0.05 (median 0.063, range = 0.17) after 3 months (*P* < 0.001). The difference between baseline and CVI after three months was not statistically significant (*P*=0.61) (Table 2, Figure 2).

Table 2 and Figure 2 show each visit's luminal, stromal, and total choroidal area. While the stromal component showed a significant decrease between the baseline and first-month values (1.16 ± 0.29 to 1.01 ± 0.27, *P*=0.03), the luminal area decrease mostly occurred between months 1 and 3 (2.03 ± 0.28 to 1.91 ± 0.23, *P*=0.045). Total choroidal area remained unchanged over the study period.

Subfoveal choroidal thickness (SFCT) was 340.8 ± 46.5 microns at baseline, 335.4 ± 42.4 microns after one month, and 341.1 ± 46.4 microns after three months. Regarding choroidal thickness, the changes were statistically insignificant (*P* values are shown in Table 2).

### 3.3. Reliability Results

To assess the intrarater agreement, Cronbach's alpha for baseline CVI was 0.90. Similarly, Cronbach's alpha was 0.81 for the agreement between two separate graders. Similarly, Cronbach's alpha for baseline choroidal thickness was 0.92 and 0.89, representing intrarater and interrater agreements, respectively.

## 4. Discussion

The present study demonstrated that, in patients with COVID-19, CVI significantly increased after one month and then reached the baseline measures 3 months after symptom initiation. At the same time, the changes in choroidal thickness were not significant within the study period. Similar to the choroid, the thickness of different macula regions, including fovea and para- and perifovea, did not significantly change over the disease course.

Regarding the changes in choroidal structures, the stromal area demonstrated the most prominent changes between the recovery from the disease and the first month. In contrast, the luminal area demonstrated the most notable difference between the first and third months. It is possible that extravasation of the serum and subsequent stromal edema starts upon the active disease phase and more rapidly resolves rather than the dilation of the lumens. However, the variability of changes in stroma and lumen in a normal population can further explain the observed changes in the COVID-19 patients.

There is a possible mechanism to explain the longitudinal changes observed in CVI. Although ischemia can be the consequence of inflammation, there are two main categories of hypotheses, including the ischemic versus the inflammatory changes caused by the disease [23]. There could be a similar pathogen responsible in both categories. For instance, hypoxemia in patients with COVID-19 can cause both ischemic and inflammatory changes. The same is true for the direct invasion by the virus and immune-mediated inflammation. Therefore, the present study proposes that the changes in choroidal vasculature are mainly the result of inflammatory changes rather than ischemic events. The reversible nature of inflammation could explain the return of CVI measures to the baseline. However, the exact pathogenesis needs to be further elucidated. Another finding that supports this hypothesis is the lack of changes in outer retinal thickness three months after the disease onset. One could expect the ischemia of the outer retina due to ischemic changes in the choriocapillaris.

Regarding retinal changes in patients with COVID-19, our results are in line with previous studies. Pirragla and associates [6] investigated retinal changes in acute COVID-19 using retinal images during hospitalization. The authors concluded the absence of retinal involvement in 46 hospitalized COVID-19 patients. Savastano and associates [9] demonstrated no changes in OCT-A and OCT parameters in COVID-19 patients 1 month after hospital discharge. Regarding microvascular abnormalities, Zapata et al. [24] and Abrishami et al. [25] reported significant macular VD reduction in patients with moderate to severe COVID-19 and patients who recovered from COVID-19, respectively. The difference between these studies and the present study could be related to the use of OCT-A in detecting microvascular abnormalities since the OCT-A can show more subtle changes than OCT.

Regarding choroidal changes in patients with COVID-19, the present study is the first longitudinal study that reports the choroidal changes in different intervals. We found no difference in SFCT, nasal and temporal thickness over the study period. Due to the lack of a control group, we can only speculate that choroidal thickness did not change over three months after COVID-19. However, if there would be any changes compared to the healthy controls, the change direction remains unknown in the present study. In the previous research by our team [11], SFCT was reported in 34 patients with definite COVID-19 diagnosis, and the increased SFCT (380.3 ± 12.40 µm) in comparison to the normal population (310.7 ± 57.5 µm) has been observed. The difference between the SFCT in the present study (340.8 ± 46.5 µm) and our previous report could be attributed to the point that the SD-OCT device was different from the previous report by our team (Heidelberg vs. AngioVue).

In a recent preprint, Kocamis and associates [10] investigated the choroidal alterations in patients with COVID-19 and demonstrated a lower amount of CVI in patients with COVID-19 than healthy control eyes. Despite the apparent difference between the present study results, our results cannot be compared with the mentioned study for several reasons. First, we did not compare the CVI with the healthy subject upon the disease onset. Second, Kocamis and associates did not report CVI changes in follow-up intervals. There is a possibility of CVI decrease at baseline due to hypoxic changes of the choroidal vasculature. However, the measured values return to normal or even increase within the inflammatory phases of the disease. Third, the exact period between the disease onset and image acquisition is unclear in their study. We believe that the disease period seriously affects the choroidal vascularity and CVI measurements.

Considering the pathophysiology of COVID-19, SARS-CoV-2 enters the host cells via the receptors of angiotensin-converting enzyme (ACE) type 2 with the additional help of transmembrane serine protease 2 (TMPRSS2) as the essential protease [26]. Despite the identification of ACE2 receptors in aqueous humor, retina, and retinal pigmented epithelium (RPE) of humans, the very possibility of ocular infection is unknown [27]. The amount of viral load and receptor expression and the necessity of coexpression of both ACE2 and TMPRSS2 make the ocular tissue a low-risk site of infection [27]. Therefore, the direct injection of the virus to the choroidal vasculature is a less possible mechanism rather than the role of inflammation and hypoxemia.

In a recent meta-analysis, pulmonary vascular enlargement has been investigated as a diagnostic criterion in CT scans of patients with COVID-19. The findings from 22 studies and 1969 patients with pneumonia were pooled together. Despite the considerable heterogeneity among the studies, vascular enlargement was observed in CT scans of two-thirds of COVID-19 pneumonia [28]. The exact mechanism of vascular enlargement in the lung of COVID-19 patients is unclear. However, immune reaction of the endothelium has been proposed as the underlying cause [28]. However, the comparison of choroidal vasculature with pulmonary capillaries is not directly possible because of the different regulatory mechanisms governed in each tissue. Still, the enlargement of choroidal capillaries could be considered as the background event of increased CVI.

The present study is limited by the small sample size and lack of healthy controls in each follow-up. However, the longitudinal evaluation of the choroidal structure, the use of CVI as a biomarker in the choroid, and the simultaneous assessment of retinal structures are among the strengths of the present study.

In conclusion, the choroidal vascularity index increases in patients with COVID-19 1 month after the disease onset but the increased level returns to the baseline values after 3 months. Explaining the choroidal changes, there might be a more prominent role for inflammation rather than ischemic changes. Larger comparative studies are needed to confirm the results of the present study.

In summary, in COVID-19, the choroidal vascularity index increases 1 month after the disease onset, but the increased level returns to baseline values after 3 months. The stromal component demonstrates more rapid alterations compared to lumen.

## Figures and Tables

**Table 1:** Retinal parameter over the study period.

		Baseline thickness (*µ*m)	1st month thickness (*µ*m)	3rd month thickness (*µ*m)	*P* ^*∗*^
Fovea	Full retina	249.7 ± 13.6	251.5 ± 14.8	246.7 ± 33.4	*T* = 0.43
	Inner retina	68.3 ± 8.1	69.2 ± 8.5	70.1 ± 7.3	*T* = 0.72
	Outer retina	181.5 ± 8.2	181.9 ± 8.1	182.8 ± 8.0	*T* = 0.56

Parafovea full	Whole	308.6 ± 12.8	310.5 ± 12.9	313.9 ± 13.9	*T* = 0.16
	Sup. hemisphere	309.9 ± 13.1	310.7 ± 12.9	313.1 ± 13.7	*T* = 0.058
	Inf. hemisphere	308.8 ± 13.2	309.1 ± 12.6	311.9 ± 13.1	*T* = 0.06
	Temporal	300.3 ± 12.2	301.0 ± 11.8	303.5 ± 12.5	*T* = 0.18
	Superior	314.5 ± 13.1	315.5 ± 11.1	317.3 ± 14.2	*T* = 0.17
	Nasal	311.5 ± 11.9	312.2 ± 14.7	315.4 ± 14.8	*T* = 0.08
	Inferior	311.4 ± 13.2	311.1 ± 12.5	313.6 ± 13.2	*T* = 0.21

Inner retina	Whole	129.3 ± 7.4	128.8 ± 7.6	128.8 ± 7.9	*T* = 0.19
	Sup. hemisphere	129.2 ± 6.8	129.3 ± 8.1	128.3 ± 8.1	*T* = 0.21
	Inf. hemisphere	128.9 ± 7.9	127.8 ± 8.3	127.9 ± 8.2	*T* = 0.21
	Temporal	121.1 ± 7.03	119.3 ± 7.4	119.9 ± 7.0	*T* = 0.12
	Superior	133.1 ± 7.1	134.0 ± 8.9	132.3 ± 8.9	*T* = 0.14
	Nasal	129.5 ± 9.9	129.9 ± 10.3	129.1 ± 10.2	*T* = 0.65
	Inferior	132.3 ± 8.02	131.1 ± 8.5	131.1 ± 8.7	*T* = 0.23

Outer retina	Whole	179.5 ± 6.7	181.3 ± 7.2	184.3 ± 8.1	*T* = 0.12
	Sup. hemisphere	180.8 ± 8.1	181.7 ± 6.7	184.8 ± 7.9	*T* = 0.18
	Inf. hemisphere	179.8 ± 7.5	181.2 ± 8.0	183.9 ± 8.8	*T* = 0.08
	Temporal	179.1 ± 7.4	181.7 ± 7.2	183.4 ± 7.8	*T* = 0.08
	Superior	181.4 ± 8.4	181.5 ± 6.5	185.1 ± 8.5	*T* = 0.07
	Nasal	182.0 ± 9.7	182.5 ± 8.8	186.3 ± 10.6	*T* = 0.06
	Inferior	179.0 ± 7.9	180.0 ± 8.3	182.6 ± 9.8	*T* = 0.09

Perifovea full	Whole	283.3 ± 10.1	282.6 ± 11.8	287.7 ± 11.3	*T* = 0.16
	Sup. hemisphere	286.9 ± 11.3	286.5 ± 11.1	289.1 ± 11.2	*T* = 0.18
	Inf. hemisphere	282.3 ± 11.6	289.2 ± 10.5	283.7 ± 11.9	*T* = 0.59
	Temporal	274.4 ± 9.9	277.9 ± 9.7	277.4 ± 9.9	*T* = 0.14
	Superior	286.5 ± 11.3	288.5 ± 10.4	288.6 ± 11.3	*T* = 0.21
	Nasal	300.1 ± 14.8	301.5 ± 14.2	301.3 ± 14.3	*T* = 0.24
	Inferior	277.5 ± 11.7	278.6 ± 12.8	278.4 ± 11.9	*T* = 0.37

Inner retina	Whole	113.7 ± 5.1	112.8 ± 5.9	113.4 ± 5.9	*T* = 0.19
	Sup. hemisphere	113.8 ± 5.2	114.9 ± 5.9	113.3 ± 7.8	*T* = 0.24
	Inf. hemisphere	113.9 ± 5.0	113.4 ± 6.4	111.9 ± 6.3	*T* = 0.28
	Temporal	108.3 ± 4.3	108.1 ± 4.6	107.3 ± 4.4	*T* = 0.22
	Superior	112.5 ± 4.9	113.8 ± 5.9	113.1 ± 12.9	*T* = 0.27
	Nasal	123.0 ± 8.1	123.9 ± 8.7	120.1 ± 9.5	*T* = 0.18
	Inferior	111.3 ± 5.7	110.9 ± 6.5	109.9 ± 6.2	*T* = 0.11

Outer retina	Whole	170.1 ± 6.9	171.3 ± 6.6	174.4 ± 8.4	*T* = 0.12
	Sup. hemisphere	172.9 ± 8.5	174.4 ± 6.4	175.9 ± 10.2	*T* = 0.08
	Inf. hemisphere	168.5 ± 7.1	170.7 ± 7.4	171.9 ± 8.5	*T* = 0.09
	Temporal	166.0 ± 7.3	169.7 ± 6.9	170.0 ± 7.3	*T* = 0.09
	Superior	173.8 ± 7.6	174.7 ± 6.2	175.4 ± 15.1	*T* = 0.11
	Nasal	177.0 ± 9.7	177.7 ± 9.3	181.1 ± 12.0	*T* = 0.21
	Inferior	166.3 ± 7.2	167.7 ± 7.6	168.9 ± 8.5	*T* = 0.22

^*∗*^Based on the generalized estimating equation (GEE). SD: standard deviation.

**Table 2:** Choroidal parameter over the study period.

		Baseline	1st month	3rd month	*P* value ^*∗*^	Pairwise comparison¥
Choroidal thickness (*µ*m)	SFCT	340.8 ± 46.5	335.4 ± 42.4	341.1 ± 46.4	0.54	
	Nasal	335.9 ± 49.7	332.1 ± 47.3	333.4 ± 49.2	0.47	
	Temporal	329.8 ± 50.6	330.03 ± 44.5	333.5 ± 51.2	0.77	

CVI		0.64 ± 0.05	0.67 ± 0.05	0.63 ± 0.04	0.023	0, 1 (0.012) 1, 3 (0.01)

Choroidal structural components	Luminal area (mm2)	2.03 ± 0.31	2.03 ± 0.28	1.91 ± 0.23	0.045	1, 3 (0.023) 2, 3 (0.045)
	Stromal area (mm2)	1.16 ± 0.29	1.01 ± 0.27	1.11 ± 0.24	0.006	0, 1 (0.03) 1, 3 (0.05)
	Total area (mm2)	3.19 ± 0.55	3.04 ± 0.45	3.02 ± 0.38	0.88	

^*∗*^Based on GEE; CVI: choroidal vascularity index. SD: standard deviation. SFCT: subfoveal choroidal thickness. ¥Bonferroni method was used to adjust for the multiple comparisons.

## Data Availability

The data used to support the findings of this study are available from the corresponding author upon request.

## References

[1] Li Q., Guan X., Wu P. (2020). Early transmission dynamics in Wuhan, China, of novel coronavirus–infected pneumonia. *New England Journal of Medicine*.

[2] Gupta A., Madhavan M. V., Sehgal K. (2020). Extrapulmonary manifestations of COVID-19. *Nature Medicine*.

[3] Invernizzi A., Torre A., Parrulli S. (2020). Retinal findings in patients with COVID-19: results from the SERPICO-19 study. *EClinicalMedicine*.

[4] Landecho M. F., Yuste J. R., Gándara E. (2021). COVID-19 retinal microangiopathy as an in vivo biomarker of systemic vascular disease?. *Journal of Internal Medicine*.

[5] Lani-Louzada R., Ramos C. d. V. F., Cordeiro R. M., Sadun A. A. (2020). Retinal changes in COVID-19 hospitalized cases. *PLoS One*.

[6] Pirraglia M. P., Ceccarelli G., Cerini A. (2020). Retinal involvement and ocular findings in COVID-19 pneumonia patients. *Scientific Reports*.

[7] Marinho P. M., Marcos A. A. A., Romano A. C., Nascimento H., Belfort R. (2020). Retinal findings in patients with COVID-19. *The Lancet*.

[8] Vavvas D. G., Sarraf D., Sadda S. R. (2020). *Concerns about the Interpretation of OCT and Fundus Findings in COVID-19 Patients in Recent Lancet Publication*.

[9] Savastano M. C., Gambini G., Cozzupoli G. M. (2021). Retinal capillary involvement in early post-COVID-19 patients: a healthy controlled study. *Graefe’s Archive for Clinical and Experimental Ophthalmology*.

[10] Kocamiş Ö, Temel E., Hizmali L., Aşıkgarip N., Örnek K., Sezgin F. M. (2021). Structural alterations of the choroid evaluated using enhanced depth imaging optical coherence tomography in patients with coronavirus disease. *The Arquivos Brasileiros de Oftalmologia*.

[11] Abrishami M., Daneshvar R., Shoeibi N. (2021). Spectrum disorder findings in patients with coronavirus disease. *Case Reports in Ophthalmology Medicine*.

[12] Bertoli F., Veritti D., Danese C. (2020). Ocular findings in COVID-19 patients: a review of direct manifestations and indirect effects on the eye. *Journal of ophthalmology*.

[13] Casagrande M., Fitzek A., Püschel K. (2020). Detection of SARS-CoV-2 in human retinal biopsies of deceased COVID-19 patients. *Ocular Immunology and Inflammation*.

[14] Fu J., Zhou B., Zhang L. (2020). Expressions and significances of the angiotensin-converting enzyme 2 gene, the receptor of SARS-CoV-2 for COVID-19. *Molecular Biology Reports*.

[15] Sun K., Gu L., Ma L., Duan Y. (2021). Atlas of ACE2 gene expression in mammals reveals novel insights in transmission of SARS-Cov-2. *Heliyon*.

[16] Agrawal R., Gupta P., Tan K. A., Cheung C. M, Wong T. Y, Cheng C. Y (2016). Choroidal vascularity index as a measure of vascular status of the choroid: measurements in healthy eyes from a population-based study. *Scientific Reports*.

[17] Agrawal R., Salman M., Tan K.-A. (2016). Choroidal vascularity index (CVI) - a novel optical coherence tomography parameter for monitoring patients with panuveitis?. *PLoS One*.

[18] Tan R., Agrawal R., Taduru S., Gupta A., Vupparaboina K., Chhablani J. (2018). Choroidal vascularity index in retinitis pigmentosa: an OCT study. *Ophthalmic Surgery, Lasers and Imaging Retina*.

[19] Wei X., Ting D. S. W., Ng W. Y., Khandelwal N., Agrawal R., Cheung C. M. G. (2017). Choroidal vascularity index. *Retina*.

[20] Agrawal R., Ding J., Sen P. (2020). Exploring choroidal angioarchitecture in health and disease using choroidal vascularity index. *Progress in Retinal and Eye Research*.

[21] Wong I. Y., Koizumi H., Lai W. W. (2011). Enhanced depth imaging optical coherence tomography. *Ophthalmic Surgery, Lasers and Imaging Retina*.

[22] Sonoda S., Sakamoto T., Yamashita T. (2014). Choroidal structure in normal eyes and after photodynamic therapy determined by binarization of optical coherence tomographic images. *Investigative Opthalmology & Visual Science*.

[23] Wijeratne T., Gillard Crewther S., Sales C., Karimi L. (2021). COVID-19 pathophysiology predicts that ischemic stroke occurrence is an expectation, not an exception—a systematic review. *Frontiers in Neurology*.

[24] Zapata M. Á, García S. B., Sánchez A. (2020). Retinal microvascular abnormalities in patients after COVID-19 depending on disease severity. *British Journal of Ophthalmology*.

[25] Abrishami M., Emamverdian Z., Shoeibi N. (2021). Optical coherence tomography angiography analysis of the retina in patients recovered from COVID-19: a case-control study. *Canadian Journal of Ophthalmology*.

[26] Senapati S., Banerjee P., Bhagavatula S. (2021). Contributions of human ACE2 and TMPRSS2 in determining host–pathogen interaction of COVID-19. *Journal of Genetics*.

[27] Schnichels S., Rohrbach J. M., Bayyoud T. (2020). Can SARS-CoV-2 infect the eye? An overview of the receptor status in ocular tissue. *Ophthalmologe, Der*.

[28] Lv H., Chen T., Pan Y., Wang H, Chen L, Lu Y (2020). Pulmonary vascular enlargement on thoracic CT for diagnosis and differential diagnosis of COVID-19: a systematic review and meta-analysis. *Annals of Translational Medicine*.

